# The potential of nanomedicine therapies to treat neovascular disease in the retina

**DOI:** 10.1186/2040-2384-2-21

**Published:** 2010-10-08

**Authors:** Krysten M Farjo, Jian-xing Ma

**Affiliations:** 1 Department of Physiology, University of Oklahoma Health Sciences Center, Oklahoma City, OK 73104 USA

## Abstract

Neovascular disease in the retina is the leading cause of blindness in all age groups. Thus, there is a great need to develop effective therapeutic agents to inhibit and prevent neovascularization in the retina. Over the past decade, anti-VEGF therapeutic agents have entered the clinic for the treatment of neovascular retinal disease, and these agents have been effective for slowing and preventing the progression of neovascularization. However, the therapeutic benefits of anti-VEGF therapy can be diminished by the need for prolonged treatment regimens of repeated intravitreal injections, which can lead to complications such as endophthalmitis, retinal tears, and retinal detachment. Recent advances in nanoparticle-based drug delivery systems offer the opportunity to improve bioactivity and prolong bioavailability of drugs in the retina to reduce the risks associated with treating neovascular disease. This article reviews recent advances in the development of nanoparticle-based drug delivery systems which could be utilized to improve the treatment of neovascular disease in the retina.

## Introduction

Retinopathy of prematurity (ROP), diabetic retinopathy (DR), and age-related macular degeneration (AMD), are the leading causes of blindness in infants, working-age adults, and the elderly, respectively [1-4]. These retinal diseases of varying etiology culminate with the development of pathogenic neovascularization, which disrupts retinal structure and function, causing irreversible vision loss. Although we understand much of the molecular mechanisms of neovascularization and have identified molecular targets and effective treatment options, sustaining safe and efficient drug delivery to the retina remains the primary obstacle to effectively treating neovascular disease in the retina. This is due to the inherent, isolated nature of the eye and the retina, which possesses a blood-retinal-barrier (BRB) to limit the diffusion of substances from the blood into the retina [5,6].

**Figure 1:**
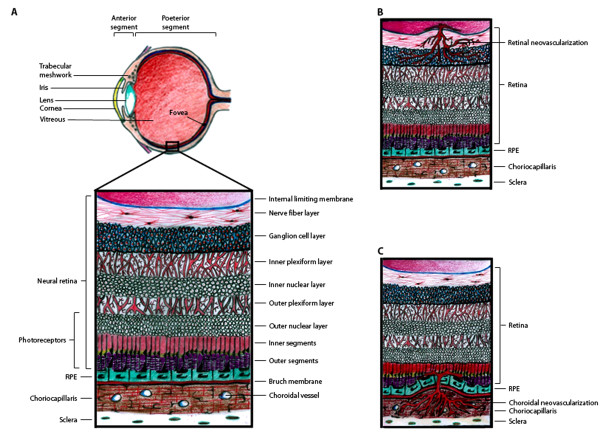
**Schematic representation of the retina and sites of pathogenic neovascularization**. **(A) **Illustration of the eye, with the anterior segment consisting primarily of the lens, iris, and cornea and the posterior segment consisting primarily of the vitreous and retina. The small box highlights the location of the retinal tissue which lines the back of the eye and is diagramed in more detail. The retina is stratified into highly ordered layers as labeled in the picture. **(B) **Retinal neovascularization occurs when retinal capillaries pass through the inner limiting membrane and invade the retinal tissue, primarily in the ganglion cell layer. **(C) **Choroidal neovascularization occurs when choroidal capillaries pass through Bruch's membrane and invade the RPE and subretinal space.

The retina consists of seven layers of neuronal cells, including the photoreceptor cells which convert light stimuli into electrical signals that are sent through the other retinal neuronal cells to the optic nerve in order for visual perception to occur (Figure 1A). Adjacent to the photoreceptor cells, there is a monolayer of retinal pigment epithelial (RPE) cells. On the other side of the RPE cell monolayer, there is a basement membrane of extracellular matrix molecules known as Bruch's membrane, which separates the RPE from the choroidal vasculature. There are two levels of the BRB, the outer BRB (oBRB), which is formed by intercellular tight junctions in the RPE monolayer to restrict the passage of molecules from the choroidal blood supply into the neural retina, and the inner BRB (iBRB), which is formed by a monolayer of specialized non-fenestrated endothelial cells that form tight junctions within the retinal capillaries to prevent widespread diffusion of substances into the retina [5,6]. The BRB is a major obstacle for drug delivery to treat retinal diseases [7]. Systemic drug dosing, via oral, intravenous, subcutaneous, or intraperitoneal administration is not very effective for drug delivery to the retina, since only 1-2% of the drug reaches the RPE and neural retina [8,9]. Likewise, topical administration of drugs on the ocular surface in the form of eye drops or ointments is also inefficient for drug delivery to the retina. Thus, intravitreal (IVT) injection is most commonly used for drug administration to treat retinal disease. Although IVT injection can efficiently deliver drugs to the retina and RPE, prolonged treatment for chronic diseases often requires repeated injections, which can lead to severe complications, such as infections and retinal detachment.

DR and AMD are chronic, progressive diseases that lead to neovascularization within the retina. Therapeutic agents can slow and prevent the progression of neovascularization in DR and AMD, but the therapeutic benefits can be diminished by inefficient drug delivery and the limited duration of drug bioavailability, which requires prolonged treatment regimens of repeated IVT injections [10,11]. Thus, improved drug delivery systems must be developed to treat neovascularization in DR and AMD. This article reviews the latest approaches to target and treat neovascular disease in the retina, with specific emphasis on recent preclinical studies in animal models and early-phase clinical trials aimed at developing nanomedicine modalities for more efficient and sustained delivery of therapeutic agents to the retina.

## Cellular and Molecular Mechanisms of Pathogenic Neovascularization in the Retina

There are two types of neovascularization that occur in the retina and cause vision loss: retinal neovascularization (RNV) in which new vessels sprout from the retinal capillaries and invade the vitreous and neural retinal layers, and choroidal neovascularization (CNV) in which new vessels sprout from the choroidal vasculature and invade the subretinal space (Figure 1B and 1C). RNV can occur in both ROP and proliferative DR [1-3,12], whereas CNV can occur in patients with AMD [13,14]. Although RNV and CNV originate from different vascular networks and invade different layers of the retina, shared molecular mechanisms promote the progression of both.

In the pathogenesis of AMD, RPE cell function is impaired, which causes toxic cellular debris to accumulate intracellularly and underneath the basal surface of the RPE cell layer in the Bruch's membrane. Subsequently, RPE cell death can occur in patches known as geographic atrophy, and compromise the oBRB. At sites of geographic atrophy, ischemia and inflammation can promote CNV into the subretinal space. The newly forming blood vessels are leaky and cause inflammation and damage, resulting in photoreceptor cell death and permanent vision loss.

In DR, high blood glucose levels cause oxidative stress in endothelial cells, which results in cellular metabolic dysfunction and leads to retinal capillary basement membrane thickening. This initiates pericyte and endothelial cell death, resulting in breakdown of the iBRB. The loss of retinal capillary function causes vascular leakage and inflammation, as well as retinal ischemia, which promotes RNV and leads to irreversible vision loss.

ROP occurs in premature infants who are exposed to relative hyperoxia before the angiogenic phase of retinal development is complete [12]. This is problematic, since the angiogenic phase of retinal development is normally driven by hypoxia *in utero *[12]. Thus, normal angiogenic retinal development is disturbed in ROP, causing vaso-obliteration and the formation of a largely avascular retina [12]. In the absence of an adequate blood supply, the avascular retina is ischemic, which promotes destructive RNV, and can lead to retinal detachment and the formation of scar tissue, resulting in permanent vision loss [12].

Retinal ischemia is a common component of the pathogenesis of both CNV and RNV. Ischemia causes cellular hypoxia, which activates cellular signaling pathways to up-regulate the expression of angiogenic stimulators, such as vascular endothelial growth factor (VEGF) [15]. VEGF is a secreted glycoprotein with potent pro-angiogenic activity. VEGF binds to VEGF receptors (VEGFR) on endothelial cells to stimulate cell proliferation and migration. Numerous studies have shown that VEGF is up-regulated during the pathogenesis of CNV and RNV, and that VEGF is a key mediator of CNV and RNV pathogenesis [15].

### Disrupted Balance of Angiogenic and Anti-Angiogenic Factors in RNV and CNV

The normal retina expresses a low amount of VEGF in the RPE, and high levels of angiogenic inhibitors, such as pigment epithelium-derived factor (PEDF) [16,17]. PEDF is a secreted glycoprotein that belongs to the serine proteinase inhibitor (SERPIN) family, but does not have SERPIN activity. PEDF has potent anti-angiogenic activity and counteracts the effects of VEGF [18]. Thus, in the normal retinal homeostasis, the balance between pro- and anti-angiogenic factors tips in favor of angiogenic inhibition. This balance is disrupted during the pathogenesis of both CNV and RNV, as retinal ischemia promotes the up-regulation of VEGF expression and the down-regulation of PEDF expression, creating an increased VEGF/PEDF ratio that strongly promotes angiogenic stimulation during CNV and RNV [16,17,19].

Therapeutic interventions that either decrease the VEGF/PEDF ratio or inhibit VEGF activity can significantly inhibit CNV and RNV progression [11,18,20]. In rodent models, IVT injection of either recombinant PEDF protein or an adeno-associated viral plasmid expressing PEDF effectively decreases the VEGF/PEDF ratio and significantly reduces RNV and CNV [18,21]. VEGF is the primary angiogenic stimulator in CNV and RNV, which has been highlighted by the clinical success of therapeutic agents that inhibit VEGF activity for the treatment of AMD and DR [11,20]. However, anti-VEGF therapies have reduced efficacy during long-term treatment regimens. In a clinical study of patients with AMD, the efficacy of a single IVT injection of the anti-VEGF antibody Avastin^® ^decreased to 50% of the initial dose response by the third IVT injection dose [22]. This phenomenon, known as tachyphylaxis, can contribute to the recurrence of neovascularization after anti-VEGF therapy.

Other angiogenic stimulators, such as platelet-derived growth factor (PDGF) and fibroblast growth factor (FGF) can also promote the pathogenesis of CNV and RNV, but therapeutically targeting either PDGF or FGF alone is not as effective as targeting VEGF activity; Nevertheless, studies suggest that combining PDGF or FGF inhibitors with VEGF inhibitors can have synergistic therapeutic effects in reducing the pathogenesis of CNV [23,24]. In the future, combining therapies that target more than one angiogenic factor are likely to improve clinical outcome for AMD and DR patients,

In addition to PEDF, other angiogenic inhibitors are also expressed in the retina/RPE and have been implicated to play a role in the pathogenesis of CNV and RNV. For instance, another SERPIN family member, SERPINA3K, is an angiogenic inhibitor expressed in the normal retina that is down-regulated during the pathogenesis of RNV in DR [25]. In a rodent model of RNV, IVT injection of recombinant SERPINA3K protein decreased hypoxia-induced VEGF up-regulation and significantly reduced RNV and vascular leakage [26,27]. Thrombopsondins (TSPs) are a type of secreted glycoprotein expressed by endothelial cells and RPE. TSP1 and TSP2 can inhibit endothelial cell proliferation and migration *in vitro *[28]. TSP1 is expressed in human RPE, and its expression is down-regulated in AMD [19,29]. *Tsp1^-/- ^*mice have increased retinal vascular density [30], whereas overexpression of TSP1 significantly inhibits RNV in the oxygen-induced retinopathy (OIR) mouse model [31]. Conversely, one study demonstrated that TSP1 stimulates VEGF and FGF2 secretion from cultured RPE cells [32], and another study found that TSP1 is necessary for PDGFB-mediated stimulation of pericyte proliferation and migration [33]. Thus, TSPs may be considered angiogenic modulators, and not strict angiogenic inhibitors.

Several angiogenic inhibitors are generated from the proteolytic cleavage products of native proteins, which display no angiogenesis-related activity prior to cleavage. One notable example is plasminogen, a pro-enzyme that is cleaved to generate the fibrinolytic enzyme plasmin. Additional cleavage of plasmin produces peptides with anti-angiogenic activity, including angiostatin and kringle 5 (K5). Angiostatin is a 38 kDa polypeptide which contains the first four triple disulfide bond-linked loops of plasminogen known as kringle domains [34]. Systemic (subcutaneous) or IVT injection of angiostatin reduces CNV, RNV and vascular leakage in rodent models [35-37]. K5 is the fifth kringle domain of plasminogen, consisting of only 80 amino acids. K5 is more potent than angiostatin for inhibiting bFGF-stimulated endothelial cell proliferation *in vitro *(ED_50 _= 50 nM vs. 140 nM, respectively) [38]. In rodent models, IVT injection of either recombinant K5 protein or adeno-associated viral plasmid expressing K5 significantly decreases VEGF expression, increases PEDF expression, and reduces RNV [39-41].

Another group of angiogenic inhibitors, named vasoinhibins, are generated by the proteolytic cleavage of prolactin, growth hormone, or placental lactogen. Prolactin and prolactin-derived vasoinhibins are present in the retina [42], and prolactin-derived vasoinhibins can block VEGF-induced vasopermeability in rats with DR [43]. In rodent models, IVT injection of either antibodies against vasoinhibins or siRNA against prolactin causes retinal angiogenesis and vasodilation [42], whereas injection of recombinant vasoinhibin can suppress RNV [44]. These data suggest that prolactin-derived vasoinhibins are important angiogenic inhibitors in the retina.

Extracellular matrix (ECM) proteins, which are abundant in the retinal capillary basement membrane as well as the Bruch's membrane adjacent to the choriocapillaris, can also be cleaved to generate angiogenic inhibitors. The native or un-cleaved forms of these basement membrane proteins display no angiogenesis-related activity. This is intriguing, since the proteolytic digestion of the capillary basement membrane necessarily precedes angiogenic sprouting of new blood vessels. This implies that angiogenic inhibitors may be produced during early angiogenic sprouting in order to counterbalance angiogenic stimulators like VEGF and limit the extent of neovascularization. The most well-studied ECM-derived angiogenic inhibitor is endostatin, a 20 kDa C-terminal fragment derived from collagen XVIII alpha 1 (Col18α1) [45]. Endostatin is expressed in the human RPE [46], and its expression is decreased in AMD [19]. In a mouse model of laser-induced CNV, *Col18α1^-/- ^*mice developed 3-fold larger CNV lesions than wild-type mice [47]. Moreover, intraperitoneal (i.p.) injection of recombinant endostatin significantly reduced CNV lesion size [47]. Recombinant endostatin was the first endogenous angiogenic inhibitor to begin clinical trials as an anti-tumor therapy [48], and although it was non-toxic, it lacked potent efficacy as a monotherapy [48,49]. Since then, both endostatin and an N-terminally tagged version of endostatin known as Endostar, have been combined with chemotherapeutic agents to increase tumor regression in clinical trials [50]. In 2005, Endostar was approved for the treatment of non-small-cell lung cancer in China, but it has yet to gain approval from the U.S. Food and Drug Administration (FDA). Another ECM-derived angiogenic inhibitor, tumstatin, is generated from the cleavage of collagen type IV. Tumstatin binds to α_v_β_3 _integrin, which is highly expressed on the cell surface of proliferative, neovascular endothelial cells. Tumstatin can significantly inhibit endothelial cell proliferation *in vitro *[51], suggesting that it could function to reduce RNV and CNV, although the angiogenic role of tumstatin has not yet been investigated in animal models of RNV or CNV.

## Current Treatment Options for RNV and CNV

A common treatment for DR is laser-induced photocoagulation, in which a laser is used to alleviate hypoxia in the retina and attenuate RNV [52]. Although photocoagulation can stabilize vision and reduce the risk of future vision loss in many patients, there are significant risks associated with photocoagulation therapy, since the laser treatment alone can cause damage to the retina and permanently impair vision [52]. Furthermore, laser photocoagulation therapy does not stop the progression of DR in all patients. A similar, but safer laser-based method, photodynamic therapy (PDT), was the first FDA-approved therapy for the treatment of neovascular AMD. PDT utilizes a photo-activatable drug, verteporfin (Visudyne^®^, QLT Ophthalmics/Novartis AG), which is administered intravenously [53]. Vertoporfin collects in the choriocapillaris, and a low-energy laser beam is focused onto CNV lesions to activate verteporfin, which will induce blood clot formation to seal off abnormal neovascular blood vessels [53]. PDT cannot regress CNV lesions, but it can reduce the progression of CNV, although PDT must be repeated to sustain inhibition of vascular leakage [54].

A plethora of studies over the past decade have investigated the development of therapeutic agents that directly target the molecular mechanisms of angiogenesis. VEGF is the primary angiogenic stimulator in the pathogenesis of RNV and CNV [15]. Thus, several therapeutic agents have been designed to specifically inhibit VEGF activity, and such drugs have had clinical success in the treatment of DR and AMD [15]. In 2004, pegaptanib (Macugen^®^, Eyetech Inc.) was the first drug to obtain FDA approval for the treatment of CNV in AMD [55]. Macugen^® ^is a 50 kDa RNA aptamer that binds to and inhibits VEGF [11,55]. Also in 2004, a humanized monoclonal anti-VEGF antibody, bevacizumab (Avastin^®^, Genentech) was approved for anti-angiogenic therapy in cancer [56]. Avastin^® ^is still in clinical trials for the treatment of AMD and DR, but it is routinely prescribed off-label for AMD patients [11,56]. A smaller fragment of the bevacizumab antibody, ranibizumab (Lucentis^®^, Genentech) was FDA-approved specifically for the treatment of AMD in 2006, and is undergoing further clinical trials for the treatment of DR [11,57]. Several clinical trials have shown that anti-VEGF therapeutic agents are more effective than PDT in maintaining and restoring visual acuity and reducing CNV progression in patients with AMD [10,56]. Thus, other inhibitors of VEGF activity are also in development, including a soluble VEGFR mimetic, aflibercept (VEGF Trap-Eye™, Regeneron), and a siRNA that inhibits VEGF expression, bevasiranib (Cand5™, OPKO Health Inc.) [11]. The VEGF Trap-Eye™ is currently in Phase III clinical trials, and preliminary results have shown that it has been an effective treatment for CNV in AMD [58]. Clinical trials investigating the use of Cand5™ as a monotherapy were terminated in 2009 because Cand5™ therapy was less effective than Lucentis^® ^therapy; however, Cand5™ is now in a clinical trial as a combination therapy administered in conjunction with Lucentis [11].

Although these anti-VEGF therapies have been effective for slowing disease progression and reducing the risk of vision loss due to AMD and DR, these therapies are limited by the need for burdensome and risky IVT injections, which must be repeated every 4-12 weeks in order to sustain therapeutic levels of the drugs in the retina [10,11]. IVT injection can lead to vision-threatening complications, such as endophthalmitis, cataract, retinal tears, and retinal detachment [10,59]. Thus, more effective drug delivery systems are desired to circumvent the need for IVT injection or at least reduce the frequency of IVT injections to thereby improve safety and increase patient compliance and patient outcome.

## Developing Superior Therapeutic Agents with Nanotechnology

Nanotechnology offers the opportunity to create new drug delivery systems (DDS) to improve drug efficacy and safety for the treatment of neovascular disease in the retina. Nanotechnology has been defined as the design, characterization, production, and application of structures, devices, and systems by controlled manipulation of size and shape at the nanometer scale (atomic, molecular, and macromolecular scale) that produces structures, devices, and systems with at least one novel or superior characteristic or property [60]. Nanotechnology classically refers to matter in the size range of 1-100 nm, but it is often extended to include materials below 1 μm in size. The small size of nanotechnology materials could be especially useful for retinal drug delivery of systemically-administered drugs, which can be hindered by the BRB. Several studies have already demonstrated that some types of nanoparticles can cross the BRB to deliver therapeutics to the retina without exerting obvious cytotoxicity [61-63]. Furthermore, nanotechnology can be used to optimize drug formulations to increase drug solubility and alter pharmacokinetics to sustain drug release and thereby prolong bioavailability. In addition, the diverse platforms of nanotechnology can also be utilized to develop more sophisticated, cell-targeted therapies and to combine different drugs into one nanotherapeutic agent for synergistic therapeutic benefits.

Nanotechnology could be harnessed to reformulate anti-VEGF therapies for prolonged bioavailability and targeted delivery to neovascular lesions. However, nanotechnology-based DDS are in early stages of development, and reformulation of anti-VEGF therapies with nanotechnology-based DDS would require that new anti-VEGF "nanotherapies" be reevaluated for safety and efficacy in clinical trials, which is costly and time-consuming. Nevertheless, numerous preclinical studies suggest that nanotechnology-based DDS can address and overcome many of the challenges of retinal drug delivery to greatly improve therapeutic outcomes. This should encourage pharmaceutical scientists to co-develop nanotechnology-based DDS for new anti-neovascular therapeutic agents during preclinical development in order to generate superior nanotherapeutic agents for clinical trials.

### Nanoparticle Platforms for Drug Delivery Systems

There is a diverse arsenal of nanoparticle systems available for the development of both simple and sophisticated nanotherapeutic agents to target neovascular disease in the retina. Nanoparticle platforms include synthetic and natural lipid-, polymer-, polypeptide-, and polysaccharide-based systems, as well as metallic nanoparticulates, such as gold [64-67]. Lipid-based nanoparticles can be used to generate liposomes, which consist of a phospholipid bilayer membrane that encapsulates cargo molecules [68]. Since naturally-occurring phospholipids are often used to generate liposomes, they are generally found to be biocompatible, non-toxic, and non-immunogenic. Liposomes can encapsulate either hydrophobic or hydrophilic molecules with high efficiency. Several liposome-based nanoparticle DDS have been FDA-approved for clinical use [68]. However, liposomes can be somewhat unstable, and stability can be improved by generating hybrid liposome-polymer nanoparticles. The polymeric compound polyethylene glycol (PEG) is most commonly used for this purpose. PEG is the most widely used polymeric nanoparticle system, and it can greatly extend the bioavailability of therapeutic agents.

The polymers polylactide (PLA) and polyglycolide (PGA) are also widely used for nanoparticle DDS. PLA and PGA are often mixed to generate the copolymer Poly(*D,L-*lactide-*co*-glycolide) (PLGA) [69,70]. Various ratios of PLA/PGA can be utilized to generate PLGA nanoparticles which have distinct and well-characterized rates of degradation [69]. PLGA is biocompatible, biodegradable, non-toxic, and non-immunogenic, and thus, numerous PLGA-containing therapeutic agents have been approved by the FDA [71]. PLGA-based nanoparticle DDS have been extensively studied for gene therapy applications, as PLGA has been shown to mediate endo-lysosomal escape, which reduces DNA plasmid degradation and increases delivery of DNA plasmids to the nuclear compartment [72].

In recent years, polymeric dendrimers have also been developed as nanoparticle DDS. Dendrimers are globular macromolecules which contain a central core element from which highly-branched structures emanate [73]. Dendrimer branches can be extended by stepwise synthesis, which allows for precise control of dendrimer structure, molecular weight, solubility, size, and shape. Thus, dendrimers are well-defined in size and composition compared to other nanoparticle DDS [73]. In addition, natural polymers, such as polypeptides and polysaccharides can also be used for nanoparticle DDS [67]. Polypeptide-based nanoparticles are most commonly generated using either albumin or poly-L-lysine, whereas polysaccharides, such as hyaluronic acid, heparin, chitosan, and cyclodextrin, can be formulated into nanoparticles alone or in combination with lipid-based or polymer-based nanoparticle platforms [64,67,74].

Metals, such as gold, silver, and platinum, can also be used for nanoparticle DDS. Gold is most commonly used, as it is inert, non-toxic, and non-immunogenic. A recent study showed that gold nanoparticles of 20 nm can pass through the BRB and exhibit no retinal toxicity, suggesting that gold nanoparticles could be used to safely and effectively deliver therapeutic agents to the retina [62]. Interestingly, naked gold nanoparticles have intrinsic anti-angiogenic activity. Moreover, gold nanoparticles conjugated with glycosaminoglycans have enhanced anti-angiogenic activity [75,76]. This phenomenon has also been observed in chitosan nanoparticles and sixth generation poly-L-lysine dendrimers, which possess inherent anti-angiogenic activity [77,78]. These observations warrant further investigation into the use of such nanoparticles for neovascular disease.

### Development of Nanoparticle DDS to Treat Neovascular Disease in the Retina

Promising anti-neovascular therapeutic agents include gene therapy vectors, peptide-based inhibitors, antibodies, oligonucleotide aptamers, and small molecules. Some of these therapeutic agents have been combined with nanotechnology-based DDS in preclinical studies, resulting in increased and prolonged bioavailability, enhanced cell targeting, and overall increased therapeutic benefit compared to conventional DDS in animal models. The potential applications of nanoparticle-based DDS for the treatment of retinal neovascular disease are highlighted in the following sections.

### Nanoparticles in Gene Therapy

Chronic and progressive retinal diseases, such as AMD and DR, require sustained delivery of therapeutic agents to the retina. As mentioned previously, although anti-angiogenic therapy with anti-VEGF agents has improved the treatment of AMD, these agents must be delivered to the retina by IVT injection every 4-12 weeks to maintain therapeutic benefits [10,11]. Gene therapy-based delivery of anti-angiogenic factors could theoretically provide significantly prolonged therapeutic benefits after a single IVT injection.

The development of gene therapy vectors has surged over the past 15-20 years, and gene therapy has shown both significant successes and failures in the clinic [79,80]. Viral vectors, such as recombinant adeno-associated viral vector (rAAV), have been most commonly used for gene therapy applications. However, there are significant safety concerns regarding the use of rAAV for gene therapy, as human clinical trials with rAAV have lead to oncogenesis and fatal systemic inflammation [79,81-83]. In addition to the potential for adverse immunological responses, rAAV has a limited capacity for insert DNA (< 5 kb) as well as limited cell tropism [79]. Nevertheless, recent human clinical trials in patients with Leber's congenital amourosis caused by null mutations in RPE-specific protein 65 kDa (RPE65) have demonstrated that a single IVT injection of rAAV that expresses RPE65 can mediate expression of RPE65 for up to 1.5 years and improve vision without eliciting adverse immunological responses [80,84,85]; however, a transient increase in neutralizing antibodies to the rAAV capsid protein was observed [80]. Although the rAAV-RPE65 gene therapy results are hopeful at this point, the long-term safety and efficacy remains to be determined. rAAV-mediated gene therapy in the retina has been relatively safe thus far, due to the BRB-mediated immune-privileged state of the retina, although IVT injection of rAAV vectors in rats and dogs results in rAAV transfer to the brain [86,87], suggesting that rAAV vectors should be used with caution.

As a potential treatment for CNV, a rAAV was generated to express recombinant human PEDF [21]. Periocular (scleral) injection of rAAV-PEDF resulted in increased PEDF expression in the retina, RPE, and choroid and resulted in a significant reduction in CNV lesions in mouse and pig models [21,88]. In a recent Phase I clinical trial, rAAV-PEDF was administered by a single IVT injection to patients with neovascular AMD (CNV) [89]. The injection resulted in transient intraocular inflammation and increased intraocular pressure in 25% and 21% of patients, respectively. No other adverse inflammation occurred, suggesting the gene therapy was reasonably safe. Depending on the rAAV-PEDF dosage, between 50% and 71% of patients experienced either no change or improvement in CNV lesion size at 6 months post-injection. These results provide a proof-of-concept that angiogenic inhibitors can be delivered to the retina/RPE by gene therapy vectors; however, the use of non-viral vectors could reduce or prevent the incidence of intraocular inflammation observed with rAAV injection.

Non-viral DNA vectors offer a safe alternative to rAAV-mediated gene therapy, as non-viral vectors are non-immunogenic and non-toxic. Previously, the use of non-viral vectors has been limited due to low transfection efficiency and increased susceptibility to nuclease degradation. However, novel nanotechnology-based DDS have offered new potential for the use of non-viral vectors for gene therapy applications. Non-viral DNA vectors as large as 20 kb can now be compacted into nanoparticles of less than 25 nm in diameter, which allows the DNA to pass through nuclear pores [90]. This greatly enhances the transfection efficiency of non-viral vectors, especially in post-mitotic cells which could not be transfected by conventional non-viral DNA vectors [90-92]. Moreover, nanoparticle encapsulation also prolongs vector half-life by protecting the DNA from nuclease degradation.

In an effort to develop an efficient non-viral gene therapy vector for the treatment of RNV, we recently encapsulated a non-viral K5 expression plasmid into PLGA:Chitosan nanoparticles to produce a K5 nanoparticle expression vector (K5-NP) [93]. PLGA is a biocompatible, biodegradable polymer that is FDA-approved for use in humans [70]. PLGA nanoparticles have previously been shown to interact with the endo-lysosomal membrane and escape from the endocytic pathway into the cell cytosol, which may increase the delivery of PLGA nanoparticles to the nucleus [72]. Thus, PLGA-based nanoparticles are an attractive choice for gene therapy applications. The K5-NP was administered by IVT injection into rat models of ischemia-induced RNV and streptozotocin (STZ)-induced diabetes. We found that the K5-NP mediated expression of K5 in the retina for up to 4 weeks following a single IVT injection. The K5-NP expression was primarily restricted to the ganglion cell layer, with a high level of transfection efficiency. We demonstrated that the K5-NP significantly reduced ischemia-induced RNV, and decreased vascular leakage in both STZ-induced diabetes and ischemia-induced RNV [93]. The K5-NP prevented the up-regulation of VEGF and ICAM-1 in diabetic retinas for up to 4 weeks post-injection of the K5-NP. There was no detectable toxicity associated with the K5-NP, as histological analyses demonstrated that retinal structure and thickness was unaffected by K5-NP. Furthermore, the K5-NP did not increase retinal apoptotic cells, and electroretinography analyses showed that retinal physiology was normal following K5-NP injection. These studies demonstrate how nanoparticle-based DDS can facilitate non-viral gene therapy. Moreover, the K5-NP is an example of how gene therapy and nanotechnology can be combined to generate superior nanotherapeutics for the potential treatment of neovascular disease in the retina.

Peptide carriers can be incorporated into nanoparticles to enhance cellular uptake and avoid endolysosomal trafficking of cargo molecules, which may result in increased nuclear targeting of gene therapy vectors [94-96]. Peptide carriers include natural protein transduction domains and synthetic cell-penetrating peptides, which have the ability to traverse cell membranes without the use of transporters or cell surface receptors [94]. Natural protein transduction domains include the trans-activating regulatory protein (TAT) of human immunodeficiency virus and the VP22 protein from herpes simplex virus. Based on the molecular modeling of natural protein transduction domains, synthetic cell-penetrating peptides, such as Pep-1 and Pep-2 were developed. The Pep-1 and Pep-2 peptides consist of only 21 amino acid residues and contain 3 functionally distinct domains: a hydrophobic tryptophan-rich motif for cell membrane targeting, a hydrophilic lysine-rich domain derived from the SV40 large T antigen nuclear localization sequence which facilitates intracellular delivery, and a small linker domain which includes a proline residue to allow for flexibility [94]. Pep-1 and TAT peptides have been incorporated into nanoparticles to increase cellular and nuclear uptake of cargo molecules [97-100]. TAT-conjugation was able to increase nuclear targeting of 5 nm, but not 30 nm gold nanoparticles, suggesting that TAT-mediated trafficking to the nuclear compartment is restricted by nuclear pore dimensions [97-99].

Recently, a novel nanoparticle formulation was developed which compacts DNA to generate nanoparticles which contain a single DNA plasmid [92]. These nanoparticles utilize a 30-mer polylysine peptide which terminates with a single cysteine moiety (CK30). The terminal cysteine residue facilitates covalent bond formation with 10 kDa PEG to generate PEGylated CK30 (CK30-PEG). Plasmid DNA is then mixed with CK30-PEG to generate nanoparticles, and the size and shape of the nanoparticles can be adjusted by using different lysine amine counterions. Importantly, the minor diameter of each nanoparticle can be restricted to less than 25 nm, which allows CK30-PEG nanoparticles to traffic through nuclear pores [91,101]. This likely explains how CK30-PEG DNA nanoparticles can mediate efficient gene expression in post-mitotic cell types [91,92,102]. The cellular uptake and nuclear targeting of CK30-PEG nanoparticles does not involve the endocytic pathway, but appears to be mediated at least in part by binding to nucleolin. Nucleolin is selectively expressed on the plasma membrane of specific cell types, including post-mitotic retinal cells [101,103].

To investigate the potential use of CK30-PEG nanoparticles in retinal gene therapy, a reporter DNA plasmid which expressed green fluorescent protein (GFP) under the control of the cytomegalovirus promoter was compacted into CK30-PEG nanoparticles, and administered by IVT or subretinal (SRT) injection in mice [91]. SRT injection of CK30-PEG-GFP nanoparticles produced significant GFP expression in the RPE and retina, whereas IVT injection yielded significant GFP expression in the retina. Electroretinography analyses detected no abnormalities in retinal physiology due to the CK30-PEG-DNA nanoparticle injections. Total GFP expression in the retina was dependent on the amount of CK30-PEG-DNA nanoparticles injected. More recently, CK30-PEG nanoparticles were used to deliver a DNA plasmid which expressed the gene peripherin 2 (Prph2) to the retina of *Prph2^+/- ^*mice, which have a phenotype of slow retinal degeneration [104,105]. SRT injection of CK30-PEG-Prph2 nanoparticles significantly reduced retinal degeneration in *Prph2^+/- ^*mice, and sustained elevated Prph2 gene expression for up to 4 months. These promising preclinical data suggest that CK30-PEG nanoparticles could be developed for safe and effective gene therapy in the retina. Moreover, CK30-PEG nanoparticle-mediated gene therapy was safe and effective in clinical studies in cystic fibrosis patients [106]. Thus, CK30-PEG nanoparticles could potentially be a safe and effective tool for gene therapy-based approaches to treat neovascular disease in the retina. For instance, CK30-PEG nanoparticles could be utilized to deliver compacted non-viral DNA vectors encoding anti-angiogenic factors to the retina or RPE in an effort to inhibit RNV or CNV, respectively.

### Nanoparticles in Peptide and Drug Delivery

Therapeutic agents, including peptides, small molecule drugs, antibodies, and aptamers, can be formulated into nanoparticle-based DDS to improve therapeutic efficiency by increasing and prolonging bioavailability. The most simple nanotherapeutic agents are generated by condensing a therapeutic agent into nanoparticles using PEG or lipids. Thus, Macugen^® ^is considered as a nanotherapeutic, since it is formulated using PEGylation to condense the drug into nanoparticles for enhanced drug delivery. Nanoparticle-based DDS can be especially helpful for drug molecules which have limited solubility or significant cytotoxic effects, such as the small molecule drug TNP-470, an analog of fumagillin [107].

TNP-470 is a very potent and effective angiogenic inhibitor, and in early studies it was very effective as an anti-tumor agent in several types of animal tumor models [107-112]. In human clinical trials, TNP-470 appeared to be an effective therapy for Kaposi's sarcoma, non-small-cell lung cancer, renal carcinoma, and prostate tumors [107-112]; however, clinical trials were terminated when TNP-470 elicited neurotoxic effects, including short-term memory loss, seizures, dizziness, and decreased motor coordination. TNP-470 is so small that it could easily penetrate the blood-brain-barrier (BBB) to elicit these effects. Initial attempts to reformulate TNP-470 to block penetration of the BBB resulted in a drug formulation with very transient bioavailability [113]. Recently, a nanotechnology-based DDS was developed for TNP-470 in which TNP-470 is conjugated to a di-block copolymer of monomethoxy-PEG-PLA, which self-assembles into nanomicilles of approximately 20 nm diameter [114]. This new formulation, named Lodamin, can be orally administered to effectively treat melanoma and lung cancer in animal models, with no evidence of BBB penetration or neurotoxicity. An ongoing preclinical study is evaluating the effects of Lodamin in a laser-induced CNV mouse model [115]. Lodamin was administered either by a daily oral dose of 15 mg/kg body weight, or as a single IVT injection of 100 μg or 300 μg. Therapeutic outcome was assessed at 14 days post-IVT injection or on the fourteenth consecutive day of daily oral treatment. Oral dosing was nearly as effective as a single IVT injection, as both oral dosing and IVT injection resulted in significantly reduced VEGF levels and a 70- 75% regression of CNV lesion size [115]. Thus, Lodamin is an example of how a small molecule anti-angiogenic drug can be reformulated with very simple nanotechnology-based DDS to alter drug pharmacokinetics and thereby greatly enhance therapeutic benefits and reduce toxic side effects.

A PLGA nanoparticle formulation of PEDF peptide was recently evaluated as a therapeutic agent in a mouse model of retinal ischemia [116]. The PLGA-PEDF nanoparticles were directly compared to treatment with PEDF peptide alone. Retinal ischemia rapidly induces retinal ganglion cell (RGC) death, and leads to thinning of the retina as apoptosis occurs in other retinal cell layers. IVT injection of either PLGA-PEDF nanoparticles or PEDF peptide alone significantly reduced RGC cell death; however, the PLGA-PEDF nanoparticles were significantly more effective. Furthermore, the PLGA-PEDF nanoparticles provided enhanced protection against RGC apoptosis for up to 7 days post-injection, whereas the PEDF peptide alone was only effective for up to 2 days. This study highlights how nanoparticle formulations can enhance and prolong the efficacy of a peptide-based drug. Furthermore, this suggests that a PLGA-PEDF peptide nanoparticle formulation could be therapeutically effective in treating retinal neovascular disease.

### Nanoparticles for Targeted Drug Delivery

Nanoparticle carriers can greatly increase cell tropism and cell transfection efficiency; however, this can increase the non-specific uptake by non-target cells, including engulfing macrophages, which may result in decreased drug delivery to the target cell populations and increased drug side effects. Thus, modifying nanoparticles with cell-specific targeting agents can greatly enhance drug efficacy and reduce aberrant side effects. The nature of the nanoparticle formulation process allows for precise and stepwise synthesis of nanoparticle therapeutic agents. Nanoparticles that encapsulate a therapeutic agent can be constructed to carry various types of molecules on their external surface in order to target drug delivery to specific cell types. Moreover, more than one therapeutic agent can be combined into multi-layered nanoparticles to create a single nanotherapeutic agent which possesses synergistic therapeutic activity. Recent efforts to develop multi-component nanoparticle DDS which are specifically aimed at improving drug delivery to the retina and to neovascular retinal capillary endothelial cells are reviewed below.

### Targeting Neovascular Endothelial Cells

Proliferating, neovascular endothelial cells up-regulate the expression of cell surface markers, such as intercellular adhesion molecule 1(ICAM1) and α_v_β_3 _and α_v_β_5 _integrins [117]. Antibodies or peptides designed to bind to these markers can be used to target drug delivery specifically to neovascular endothelial cells. The humanized monoclonal anti-α_v_β_3 _integrin antibody known as etaracizumab (Abegrin^®^, MedImmune LLC) is already in clinical trials for cancer therapy, as it is expected to target tumor neovascularization [118,119]. Extracellular matrix proteins which bind to integrins contain arginine-glycine-apartic acid (RGD) motifs. Synthetic cyclic and linear RGD peptides can bind to α_v_β_3 _and α_v_β_5 _integrins to mediate cellular uptake [117]. Various RGD peptides have been widely used in preclinical cancer studies to target tumor vasculature, and a cyclic RGD peptide which specifically binds both α_v_β_3 _and α_v_β_5 _integrins, Cilengitide (Merck) is in clinical trials for cancer therapy [120]. An anti-ICAM1 antibody has previously been conjugated to liposomes to generate immunoliposomes with enhanced endothelial cell uptake activity *in vitro *[121]. A peptide domain cyclo(1,12)PenITDGEATDSGC (cLABL) from leukocyte function-associated antigen-1 binds with high affinity to ICAM1, and ICAM1 expressing endothelial cells have increased uptake of PLGA-PEG nanoparticles conjugated with cLABL [122]. These antibodies and peptides are examples of targeting moieties that could be combined with nanoparticle-based DDS to treat neovascular disease in the retina.

A novel integrin-binding peptide (DFKLFAVYIKYR) known as C16Y, was derived from laminin-1, and functions independently as an integrin antagonist to inhibit angiogenesis [123]. In a laser-induced CNV rodent model, IVT injection of the C16Y peptide incorporated into PLA/polyethylene oxide(PEO) nanoparticles (PLA/PEO-C16YNP) was more effective than C16Y peptide alone for reducing CNV lesion size [124]. Moreover, the PLA/PEO-C16YNP had prolonged bioavailability compared to the C16Y peptide alone, demonstrating how nanoparticle formulations can enhance the bioactivity and bioavailability of therapeutic agents designed to target neovascular endothelial cells.

An ongoing preclinical study in mice utilizes quantum dot nanocrystals (QD) to generate ICAM1-targeted nanocarriers (ITNs) by conjugating ICAM1 antibodies to the external surface of the QD [125]. ITNs specifically target proliferating, neovascular endothelial cells, which selectively express ICAM1 on their cell surface. The ITNs, which are smaller than 200 nm, bind to ICAM-1 on the neovascular ECs, which leads to clathrin-mediated endocytosis of the ITNs. The ITNs can encapsulate various therapeutic agents, such as siRNA, peptides, and small molecules, and deliver those cargoes to the neovascular endothelial cells.

In addition to the use of nanocarriers as drug delivery agents, gold nanoparticles can also be used for photothermal-induced cell killing. Gold nanoparticles can be activated by a low-energy near-infrared laser to produce heat, which causes cell damage and death. This type of photothermal therapy has previously been explored for cancer treatment [126-128]. An ongoing preclinical study is investigating the use of gold nanoparticles for the photothermal treatment of CNV in AMD. In an effort to target neovascular endothelial cells in CNV lesions, PEG-coated gold nanorods of 45 nm × 15 nm were conjugated with RGD peptides (Gold-RGD-NP) [61]. Following intravenous administration in a CNV mouse model, Gold-RGD-NPs were localized in intracellular vesicles of retinal endothelial cells. Subsequently, laser treatment specifically induced cell death of endothelial cells containing Gold-RGD-NPs, whereas nearby cells which were not laser-treated and/or did not contain gold nanoparticles remained viable. The surrounding tissue is unharmed because the low-energy near-infrared laser does not generate heat unless it is focused onto the gold nanoparticles. Moreover, the heat that is generated by the gold nanoparticles is minimal and induces apoptosis, and not rapid necrosis, of neovascular endothelial cells. Although this study is in very early preclinical stages, it indicates that gold nanoparticle-mediated photothermal therapy could be a safe and effective treatment for CNV lesions in AMD and thus warrants follow-up studies. In future studies, gold nanorods could also be conjugated with different agents to target endothelial cells, such as antibodies which bind to the neovascular endothelial cell surface markers ICAM1 or α_v_β_3 _integrin.

### Enhancing Ocular Delivery

A recent study evaluated if nanoparticles, designed to target the retina and neovascular lesions, could be administered intravenously and result in effective gene delivery to CNV lesions [63]. This study utilized the Flt23K DNA plasmid, which encodes the anti-VEGF intraceptor, a recombinant protein that includes VEGF-binding domains 2 and 3 of VEGFR-1 coupled to the endoplasmic reticulum (ER) retention signal sequence Lys-Asp-Glu-Leu (KDEL) [129]. The anti-VEGF intraceptor is designed to bind to VEGF as it is synthesized in the ER to sequester VEGF and inhibit VEGF secretion. Previous studies have shown that the Flt23K plasmid can inhibit hypoxia-induced VEGF expression and corneal neovascularization *in vivo *[129]. The most recent study encapsulated the Flt23K plasmid into PLGA nanoparticles, which were conjugated with either transferrin (Tf), RGD peptide, or both in order to facilitate delivery to retinal CNV lesions [63]. Transferrin was chosen for as a targeting peptide because the retina expresses transferrin receptors, and AMD retinas have increased transferrin uptake [130]. The Tf/RGD-targeted nanoparticles ranged in size from 380-450 nm. Within 24 hours of intravenous administration, Tf/RGD-targeted nanoparticles were delivered specifically to CNV lesions in the retina, and were not present in the contralateral control non-CNV retina. A much smaller amount of the non-targeted nanoparticle was also delivered to CNV lesions, likely due to the non-specific effect of vascular leakage. Importantly, intravenous administration did not lead to any nanoparticle detection in the brain. Nanoparticles were detected in non-retinal tissues, including the liver, lung, heart, kidney, and spleen; however, Tf/RGD-targeting did not increase nanoparticle delivery to these tissues. Thus, conjugation of Tf and/or RGD specifically increased delivery to neovascular lesions in the retina. Only Tf/RGD-functionalized nanoparticles, and not unconjugated nanoparticles, were expressed in the RPE cell layer. RGD conjugation also produced significant gene delivery to retinal endothelial cells, whereas Tf-conjugated nanoparticles were targeted more generally to the retina than to the retinal endothelial cells. Impressively, the intravenous administration of either Tf- or RGD-functionalized nanoparticles delivered enough nanoparticle to the CNV lesions to block CNV-induced up-regulation of VEGF protein in the retina and RPE-choroid and to significantly reduce the size of CNV lesions [63].

Preclinical studies have recently demonstrated that a synthetic cationic cell-penetrating peptide can facilitate the delivery of therapeutic agents, including peptides, small molecules, siRNA, and DNA, to the retina and RPE by IVT and SRT injection, respectively [95,131]. This peptide for ocular delivery (POD), [CGGG(ARKKAAKA)_4_], was modified with PEG to generate nanoparticles which compact plasmid DNA into 120-150 nm nanoparticles [96]. Subretinal injection of PEG-POD-DNA nanoparticles resulted in DNA expression in RPE cells, and was 200-fold more efficient in transfecting RPE cells than naked DNA plasmid [96]. PEG-POD-DNA plasmid has since been used to deliver a neurotrophic factor to the mouse retina, which resulted in reduced light damage-induced retinal degeneration [132]. Thus, PEG-POD nanoparticles have the potential to be adapted for the delivery of anti-neovascular therapeutic agents to the retina and RPE for the treatment of RNV and CNV.

## Conclusion

The treatment of retinal neovascular disease has been greatly improved by anti-VEGF therapies which have been developed over the past decade. However, frequent IVT injections are necessary for efficient and prolonged delivery of these therapeutic agents to the retina. Recent preclinical studies demonstrate that nanoparticle-based DDS can enhance bioactivity and prolong bioavailability of therapeutic agents in the retina. Moreover, efforts are underway to develop multi-component nanoparticle DDS to specifically target drug delivery to the retina, and more specifically to retinal neovascular endothelial cells. Thus, nanoparticle-based DDS are likely to have a large impact on the future treatment of neovascular disease in the retina.

## Abbreviations

AMD: Age-related macular degeneration; BBB: blood-brain-barrier; BRB: blood-retinal-barrier; CNV: choroidal neovascularization; DDS: drug delivery systems; DR: diabetic retinopathy; ECM: extracellular matrix; FDA: Food and Drug Administration; FGF: fibroblast growth factor; iBRB: inner blood-retinal barrier; ICAM1: intercellular adhesion molecule 1; ITNs: ICAM1-targeted nanocarriers; IVT: intravitreal; K5: kringle 5; oBRB: outer blood-retinal barrier; PDGF: platelet-derived growth factor; PDT: photodynamic therapy; PEDF: pigment epithelium-derived factor; PEG: polyethylene glycol; PGA: polyglycolide; PLA: polylactide; PLGA: Poly(*D,L-*lactide-*co*-glycolide); Prph2: peripherin 2; QD: quantum dot nanocrystals; rAAV: recombinant adeno-associated viral vector; RGC: retinal ganglion cell; RGD: arginine-glycine-apartic acid; RNV: retinal neovascularization; ROP: retinopathy of prematurity; RPE: retinal pigment epithelium; RPE65: RPE-specific protein 65 kDa; SERPIN: serine protease inhibitor; SRT: subretinal; TAT: trans-activating regulatory protein of human immunodeficiency virus; Tf: transferrin; TSP: thrombospondin; VEGF: vascular endothelial growth factor; VEGFR: vascular endothelial growth factor receptor;

## Competing interests

The authors declare that they have no competing interests.

## Authors' contributions

KF - selected publications for the review, drafted manuscript; JM - participated in design of the review, edited manuscript. All authors read and approved the final manuscript.
